# Signaling mechanisms of a water soluble curcumin derivative in experimental type 1 diabetes with cardiomyopathy

**DOI:** 10.1186/1758-5996-5-13

**Published:** 2013-03-12

**Authors:** Mohamed Talaat Abdel Aziz, Ibrahim Naguib El Ibrashy, Dimitri P Mikhailidis, Ameen Mahmoud Rezq, Mohamed Abdel Aziz Wassef, Hanan Hassan Fouad, Hanan Hosni Ahmed, Dina A Sabry, Heba Mohamed Shawky, Rania Elsayed Hussein

**Affiliations:** 1 Unit of Biochemistry and Molecular Biology, Medical Biochemistry Department, Faculty of Medicine, Cairo University, Kasr El Aini, Cairo, Egypt; 2 Internal Medicine Department, Faculty of Medicine, Cairo University, Cairo, Egypt; 3 Department of Clinical Biochemistry, Royal Free Hospital campus, University College London Medical School, University College London (UCL), London, UK; 4 Physiology Department, Faculty of Medicine, Cairo University, Cairo, Egypt

**Keywords:** Curcumin, Diabetes type I, Heme-oxygenase-I, Diabetic cardiomyopathy, p300

## Abstract

**Background:**

Curcumin exhibits anti-diabetic activities, induces heme-oxygenase-1 (HO-1) and is an inhibitor of transcriptional co-activator p300. A novel water soluble curcumin derivative (NCD) has been developed to overcome low invivo bioavailability of curcumin. We evaluated the effect of the NCD on signaling mechanisms involved in cardiomyocyte hypertrophy and studied whether its action is mediated via inducible HO-1.

**Materials and methods:**

Rats were divided into controls, controls receiving NCD, diabetic, diabetic receiving NCD, diabetic receiving pure curcumin, diabetic receiving HO inhibitor, zinc protoporphyrin IX (ZnPP IX) and diabetic receiving NCD and ZnPP IX. NCD and curcumin were given orally. After 45 days, cardiac physiologic parameters, plasma glucose, insulin, glycated hemoglobin (GHb), HO-1 gene expression and HO activity in pancreas and cardiac tissues were assessed. Gene expression of p300, atrial natriuretic peptide (ANP) and myocyte enhancer factor 2 (MEF2A and MEF2C) were studied.

**Results:**

NCD and curcumin decreased plasma glucose, GHb and increased insulin levels significantly in diabetic rats. This action may be partially mediated by induction of HO-1 gene. HO-1 gene expression and HO activity were significantly increased in diabetic heart and pancreas. Diabetes upregulated the expression of ANP, MEF2A, MEF2C and p300. NCD and curcumin prevented diabetes-induced upregulation of these parameters and improved left ventricular function. The effect of the NCD was better than the same dose of curcumin.

## Background

By the year 2025, 300 million people worldwide will have diabetes mellitus (DM) [1]. There is evidence that complications related to DM are associated with increased oxidative stress induced by generation of free radicals [2]. Antioxidant treatment which suppresses apoptosis of β-cells was shown to preserve β-cell function in diabetic mice [3].

Curcumin (1, 7 bis (4 hydroxy-3 methoxy phenol)-1, 6 heptadiene-3, 5 dione) is a yellow phenolic compound present in turmeric (*Curcuma longa*) a widely used spice in Indian cuisine. Curcumin has a number of biological applications along with a significant antioxidant activity both in vivo and in vitro [4]. Although the mechanisms involved are poorly understood, curcumin can protect against oxidative stress and induce heme oxygenase-1 (HO-1) expression, which exerts cytoprotective effects in mouse pancreatic beta-cells. These effects were mediated through the activation of Nrf2 by a PI3-kinase/Akt-mediated pathway in mouse β cells [5].

Curcumin (turmeric) exhibits therapeutic actions in DM. Abdel Aziz et al [6] reported that insulin secretion, HO-1 gene expression and HO activity were significantly increased when rat isolated islets of Langerhans were incubated in curcumin. This increase in insulin secretion was significantly decreased by incubation of islets in the HO inhibitor, stannous mesoporphyrin (SnMP) suggesting that the action of curcumin on insulin secretion from isolated islets may be, in part, mediated through increased HO-1 gene expression.

Cardiomyocte hypertrophy as well as subsequent apoptosis and focal myocardial fibrosis are structural hallmarks of diabetic cardiomyopathy and functionally manifest as defective cardiac contractility [7]. Oxidative stress, due to glucose-mediated increase in mitochondrial superoxide production, has been suggested to be of importance in several chronic diabetic complications, including cardiomyopathy [8].

DM may lead to myocardial hypertrophy in association with an upregulation of vasoactive factors such as endothelin-1 and activation of redox-sensitive transcription factors such as NF-_*κ*_B and activating protein-1 (AP-1) [8,9]. Transcription factors are regulated by transcriptional coactivators, especially those containing histone acetyltransferase (HAT) activity; p300 is the best known of such proteins [10]. Furthermore, p300 plays an important role in regulating both myocyte enhancer factor 2 (MEF2) and GATA-binding protein 4 [11]. MEF2 (MEF2A and MEF2C) is an important transcription factor in myocyte hypertrophy and is involved in mediating the hypertrophic action of glucose on cardiomyocytes [12]. MEF2 is associated with class II histone deacetylases (HDACs). Translocation of HDAC to the cytoplasm frees up MEF2, which allows for its association with HATs, like p300, leading to the transcription of effector genes [12,13].

Curcumin has been reported to be an inhibitor of p300- HAT [14] therefore; it may possess an effect in the prevention of cardiac hypertrophy and heart failure [15]. Because of the poor bioavailability of pure curcumin, a new water soluble curcumin derivative (Patent pending PCT/EG2010/000008) was used in this study.

The aim of the present work is to evaluate the effect of a novel water soluble curcumin derivative (3% curcumin content) on signaling mechanisms involved in cardiomyocyte hypertrophy and dysfunction in myocardial contractility in DM and to study whether its action is mediated via inducible HO-1.

## Materials and methods

This study was performed at the Unit of Biochemistry and Molecular Biology at The Medical Biochemistry Department, Faculty of Medicine, Cairo University, Egypt. Curcumin protein conjugate was presented free of charge to the participating researchers as a personal non-profit scientific donation to help advancement of cooperation in national medical research. The novel derivative, with 3.0% curcumin content is registered as international patent protected by the rights of “The Patent Cooperation Treaty” and are the personal property of their inventors (Patent pending PCT/EG2010/000008 Published Patent Pending, WO 2011/100984) [16].

### Experimental animals

The experiments were performed on 140 adult rats weighing 150 to 200 g obtained from an inbred colony (Curl: HEL1) at the Kasr Al-Aini animal experimental unit, Faculty of Medicine, Cairo University. These animals were kept in an environment with controlled temperature (25°C), humidity (45-75%) and 12:12 h light:dark cycle. All animals were fed ad libitum and had free access to water. The 140 rats were divided into 4 groups (Table 1). All the ethical protocols for animal treatment were followed and supervised by the animal facilities, Faculty of Medicine, Cairo University. All animal experiments received approval from the Institutional Animal Ethics Committee.

– The animals were acclimatized for 1 week before initiation of the experiment.

– DM was induced in 100 rats by a single i.p. injection of streptozotocin (STZ) dissolved in 0.1 M sodium citrate buffer, pH 4.5, at a dose of 50 mg/kg.

– After 72 h, fasting blood glucose levels were monitored and animals with blood glucose levels >200 mg/dL were considered diabetic and were distributed into the previously mentioned groups.

– Age-matched control rats were injected with an equal volume of vehicle (sodium citrate buffer).

**Table 1:** Classification of studied groups

**Groups**	
**Group 1(a)**	Control: 20 rats.
**Group 1(b)**	Pathological control: Twenty streptozotocin-induced diabetic rats
**Group 2(a)**	Twenty control rats that received a daily oral dose of the water soluble curcumin derivative (NCD) at a dose of 20 mg/kg body weight for 45 days.
**Group 2(b)**	Twenty diabetic rats that received a daily oral dose of the NCD at a dose of 20 mg/kg body weight for 45 days after the induction of DM.
**Group 3(a)**	Twenty diabetic rats that received a daily oral dose of the NCD at a dose of 20 mg/kg body weight for 45 days after induction of DM and a weekly intraperitoneal (i.p.) dose of HO-1 inhibitor, ZnPP IX (10 μmol/Kg dissolved in sodium hydroxide 0.1 N and sodium chloride 0.9% and pH adjusted to pH 7.4) for 6 weeks.
**Group 3(b)**	Twenty diabetic rats that received a weekly i.p. dose of HO-1 inhibitor, ZnPP IX (10 μmol/Kg dissolved in sodium hydroxide 0.1 N and sodium chloride 0.9% and pH adjusted to pH 7.4) for 6 weeks.
**Group 4**	Twenty diabetic rats that received a daily oral dose of pure curcumin at a dose of 20 mg/kg body weight for 45 days after induction of DM.

At the planned time of sacrifice (after 45 days):

• Ninety nine rats remained and 41 rats died with a mortality rate of (29.3%)

• Six rats from each group were used for assessment of physiological parameters: left ventricular developed pressure, left ventricular delta pressure/delta time (contractility index), systolic blood pressure and heart rate.

• Fasting blood samples were withdrawn from the retro-orbital vein of the remaining rats to assess plasma insulin, plasma glucose and glycated Hb (GHb). This was followed by sacrifice of the animals by cervical dislocation. The pancreas and heart were excised for assessment of:

– HO enzyme activity

– HO-1 gene expression by real time PCR.

– Gene expression of p300 and molecular markers of cardiac hypertrophy such as ANP, MEF2A, and MEF2C by real time PCR

### Assessment of cardiac physiological parameters

The cardiovascular system was assessed using a Langendorff apparatus. Hearts were rapidly excised and immersed in ice-cold Krebs-Henseleit solution containing heparin (5000 u) at ambient temperature (25°C), then mounted on the aortic cannula and subsequently perfused according to the Langendorff technique with Krebs-Henseleit medium at a hydrostatic pressure of 55 cm H_2_O and bubbled with a mixture of 95% O_2_ and 5% CO_2_. The time between extraction of the hearts and their attachment to the Langendorff apparatus did not exceed 2 min.

The Krebs-Henseleit solution was prepared according to the following concentrations in (g): 6.926 NaCl, 2.1 NaHCO_3_, 0.16 KH_2_PO_4_, 0.298 KCl, 0.294 MgSO_4_, 0.264 CaCl_2_ and 1.982 glucose in 1 L distilled water. A roller pump delivered the medium (flow = 15 mL/min) to an 8 μm-pore – size 47-mm-diameter filter, a membrane oxygenator, a pre-heater, and the cannula. The temperature of the heart and of the perfusion medium was maintained at 37°C by an external water bath. A latex balloon filled with saline was introduced into the left ventricle and was connected to a pressure transducer to monitor performance. We measured the intraventricular balloon volume needed to increase end diastolic pressure from 0 to 10-15 mmHg; afterwards the balloon volume was kept constant.

Mechanical performances of the left ventricle of the heart was determined by the systolic pressure, the heart rate and the peak rate of maximum left ventricular pressure rise (dp/dt) which is a good index of contractility. These mechanical performance parameters were monitored during the experiment by a balloon inserted into the left ventricle and connected to a polygraph apparatus (San-ei Instruments, Ltd.) The developed pressure was calculated (systolic pressure – diastolic pressure) [17].

### Plasma glucose estimation

Blood was collected from the retro-orbital vein into tubes containing fluoride. Plasma samples were separated by centrifugation at 3000 rpm for 10 min. Plasma glucose was measured by the glucose oxidase method [18] using a commercially available kit (Diamond, Egypt).

### Plasma insulin estimation

Plasma insulin was assayed by a commercially available Enzyme-linked immunosorbent assay (ELISA) kit supplied by DRG Diagnostics (GmbH, Germany) [19].

### Glycated hemoglobin estimation

Whole blood was collected by venipuncture from the retro orbital vein into tubes containing EDTA as anticoagulant. Glycated hemoglobin was assayed by a commercially available kit supplied by Stanbio [20].

### HO activity assay

Pancreatic and cardiac tissues were homogenized with 2.5 volume Tris–HCl buffer (10 mmol/L, pH 7.6) containing 250 mmol/L sucrose and 0.4 mmol/L phenylmethylsulfonyl fluoride. The homogenate was centrifuged at 800 g for 10 min and then at 13 500 g for 20 min to produce a mitochondrial pellet. The supernatant was withdrawn. The protein content was determined by the method of Lowry et al [21]. The activity of HO in the supernatant was determined as previously described [22].

### RNA extraction and reverse transcription

– Total RNA was extracted from pancreatic and cardiac tissues by using SV Total RNA Isolation System supplied by Promega (Promega, Madison, WI, USA) according to the manufacturer’s protocol. Extracted RNA was quantified by spectrophotometer at 260 nm

– The extracted RNA was reversed transcribed into cDNA using Reverse Transcription System kit (Cat. # A3500) supplied by Promega (Promega, Madison, WI, USA). cDNA was generated from 5 μg of total RNA extracted with 1 μl (20 pmol) antisense primer and 0.8 μl superscript AMV reverse transcriptase for 60 min at 37°C.

#### Real-time quantitative analyses for p300, ANP, MEF2A, MEF2C and HO-1 gene expression

cDNA was generated from 5 μg of total RNA extracted with 1 μl (20 pmol) antisense primer and 0.8 μl superscript AMV reverse transcriptase for 60 min at 37°C.

### Real-time quantitative analyses

The relative abundance of mRNA species was assessed using the SYBR® Green method on an ABI prism 7500 sequence detector system (Applied Biosystems, Foster City, CA). PCR primers were designed with Gene Runner Software (Hasting Software, Inc., Hasting, NY) from RNA sequences from GenBank (Table 2). All primer sets had a calculated annealing temperature of 60°. Quantitative RT-PCR was performed in duplicate in a 25 μl reaction volume consisting of 2X SYBR Green PCR Master Mix (Applied Biosystems), 900 nM of each primer and 2-3 μl of cDNA. Amplification conditions were 2 min at 50°C, 10 min at 95°C and 40 cycles of denaturation for 15 s and annealing/extension at 60° for 10 min. Data from real-time assays were calculated using the v1 · 7 Sequence Detection Software from PE Biosystems (Foster City, CA). Relative expression of p300, ANP, MEF2A, MEF2C and HO-1 was calculated using the comparative Ct method as previously described. All values were normalized to the beta actin genes and reported as fold change.

**Table 2:** Oligonucleotide primers sequence used for real-time PCR

**Gene**	**Primer sequence**
**p300**	Forward primer: 5^′^- GCTGCGCTGGAGGAAGCCAA--3^′^
**NW_047658.2**	Reverse primer: 5^′^-TTCTCGCAGCGCGCCTGAAA-3^′^
**ANP**	Forward primer: 5^′^- GCCTGAAGGGTTTTGGGCAGCA-3^′^
**NW_047727.1**	Reverse primer: 5^′^-ACCGTCACAGCCCAAGCGAC-3^′^
**MEF2A**	Forward primer: 5^′^-ACAACCTCTTGCCACGCCCG-3^′^
**NW_001084766.1**	Reverse primer: 5^′^-AGGGGAGCGCCCCATTTCCT-3^′^
**MEF2C**	Forward primer: 5^′^- GATGGGCATGACAGACGAGAAGGGA-3^′^
**NC_005101.3**	Reverse primer: 5^′^-GCCAATGACTGGGCCGACTGG-3^′^
**HO-1**	Forward primer: 5^′^-GAGCGCCCACAGCTCGACAG-3^′^
**J02722.1**	Reverse primer: 5^′^-GTGGGCCACCAGCAGCTCAG-3^′^
**β-Actin**	Forward primer: 5^′^-CCTTCCTGGGCATGGAGTCCT-3^′^
**UniSTS: 270185**	Reverse primer: 5^′^- GGAGCAATGATCTTGATCTTC-3^′^

### Statistical analysis

ANOVA test was used to test the significance between groups. Post hoc test was used to test the difference between each 2 groups using “Statistica version 8.0” (Statsoft Inc., USA) software. Data were presented as mean ± standard deviation (SD). The differences between groups were considered to be significant at *p* < 0.05 (2-sided).

## Results

### Cardiac physiological parameters

Diabetic rats showed a reduction in contractility of diabetic hearts represented by lower LV dp/dt and lower LVDP Diabetic hearts also exhibited reduced heart rate and increased systolic blood pressure.

In diabetic rats receiving the NCD or pure curcumin, left ventricular function was improved as indicated by increased heart rate, LVDP, LV dp/dt and decreased systolic blood pressure when compared with diabetic rats.

Diabetic rats receiving pure curcumin showed no significant difference in left ventricular function compared with the diabetic group receiving the NCD. Also, feeding NCD did not affect these parameters in the control group (Table 3).

**Table 3:** Physiological parameters of the studied groups

	**Control (n = 6)**	**Control + NCD (n = 6)**	**Diabetic (n = 6)**	**Diabetic + NCD (n = 6)**	**Diabetic + pure curcumin (n = 6)**	**Diabetic + ZnPP (n = 6)**	**Diabetic + ZnPP + NCD (n = 6)**
**Heart rate (beats/min)**	166^(a)^ ± 7	173^(a)^ ± 6	122^(b)^ ± 5	149^©(d)^ ± 9	139^(d)^ ± 6	118^(e)(b)^ ± 6	140^(f)(d)^ ± 5
**Systolic Blood pressure (mmHg)**	118^(a)^ ± 8	121^(a)^ ± 8	145^(b)^ ± 7	128^(a)(d)^ ± 8	130^(a)(d)^ ± 7	147^(e)(b)^ ± 6	130^(a)(f)(d)^ ± 05
**LVDP (mmHg)**	92^(a)^ ± 5.3	93.3^(a)^ ± 4.7	59^(b)^ ± 6.0	75^©(d)^ ± 4.5	70.6^(d)^ ± 5.3	57^(e)(b)^ ± 4.6	73^(f)(d)^ ± 4.1
**LV dp/dt (mmHg/sec)**	139^(a)^ ± 7.4	137.6^(a)^ ± 7.2	101.2^(b)^ ± 9.4	134.3^(a)^ ± 5.2	133^(a)(d)^ ± 5.2	94.3^(b)(e)^ ± 8.6	128.6^(a)(f)^ ± 8.5

*LV dp/dt*: left ventricular delta pressure/delta time (contractility index).

*LVDP*: Left ventricular developed pressure.

Groups that have similar letters are not significant while groups having different letters differ significantly (p < 0.05).

Results are presented as mean ± SD.

### Blood glucose, insulin levels and glycated hemoglobin levels

There was a significant increase (p < 0.001) in the mean plasma glucose level in the diabetic group compared with the control group. On the other hand the mean plasma glucose level in the control group showed no significant difference (p > 0.05) compared with the control group receiving the oral NCD. Oral NCD supplementation to diabetic rats resulted in a significant decrease (p < 0.001) in the mean plasma glucose level compared with the diabetic group. Also, pure curcumin supplementation to diabetic rats resulted in a significant decrease in plasma glucose level (p < 0.001) compared with the diabetic group, whereas, it showed no significant difference (p > 0.05) compared with diabetic group receiving the oral NCD derivative (Table 4).

**Table 4:** Biochemical parameters in studied groups

	**Control (n = 7)**	**Control + NCD (n = 10)**	**Diabetic (n = 6)**	**Diabetic + NCD (n = 8)**	**Diabetic + pure curcumin (n = 10)**	**Diabetic + ZnPP (n = 8)**	**Diabetic + ZnPP + NCD (n = 8)**
**Plasma glucose (mg/dL)**	92 ± 6.9	91 ± 5.9	307* ± 31.4	183*§ ± 21.2	192*§ ± 6.4	342*§ ± 25.5	217*§#• ± 20.5
**Plasma insulin (μg/L)**	1.10 ± 0.19	1.58*§ ± 0.36	0.47* ± 0.18	0.80*§ ± 0.22	0.71*§ ± 0.17	0.29*§ ± 0.12	0.60*#• ± 0.09
**Glycated hemoglobin (%)**	5.4 ± 0.7	5.5 ± 0.7	13.0* ± 1.0	11.3*§ ± 0.7	11.0*§ ± 1.4	12.6* ± 1.3	11.8* ± 0.5

Results are presented as mean ± SD.

*Values differ significantly from control (p < 0.05). #Values differ significantly from Diabetic + NCD (p < 0.05).

^§^Values differ significantly from diabetic (p < 0.05). • Values differ significantly from Diabetic + ZnPP (p < 0.05).

A significant decrease (p < 0.001) in the mean plasma insulin level in the diabetic group compared with the control group was detected. In addition, there was a significant increase (p < 0.01) in the mean plasma insulin level in the control group receiving the oral NCD compared with the control group. Oral NCD supplementation to diabetic rats resulted in a significant increase (p < 0.05) in the mean plasma insulin level compared with the diabetic group (Table 4).

A significant increase (p < 0.05) in the mean fasting plasma insulin level in the diabetic group receiving pure curcumin compared with the diabetic group was detected. Whereas, it showed no significant difference (p > 0.05) compared with the diabetic group receiving the oral NCD (Table 4).

There was a significant increase (p < 0.001) in the mean GHb level in the diabetic group compared with the control group. Diabetic rats supplemented with the NCD or pure curcumin showed a modest but significantly lower (p < 0.01) blood GHb level compared with the diabetic group. But, their levels were still higher than the control group. Whereas, no significant difference (p > 0.05) was detected between diabetic rats receiving the NCD or pure curcumin (Table 4).

### HO activity in heart and pancreas

There was a significant increase (p < 0.001) in the mean HO activity in the pancreas and heart of diabetic rats compared with the corresponding control groups.

NCD supplementation to the control rats significantly increased the HO-1 expression in the pancreas (p < 0.01) and heart (p < 0.001) compared with the corresponding control groups. In addition, NCD supplementation to the diabetic rats significantly increased (p < 0.001) HO-activity in the pancreas and heart compared with the corresponding control and diabetic groups (Table 5).

**Table 5:** Comparison between the HO activity levels (pmol bilirubin/mg protein/h) of studied groups

	**Control (n = 7)**	**Control + NCD (n = 10)**	**Diabetic (n = 6)**	**Diabetic + NCD (n = 8)**	**Diabetic + pure curcumin (n = 10)**	**Diabetic + ZnPP (n = 8)**	**Diabetic + ZnPP + NCD (n = 8)**
**Pancreatic tissue**	857.1 ± 156.6	1093* ± 153.7	1287.5* ± 204.8	1853.1*§ ± 208.1	1535 *§# ± 201.1	918.7§ ± 119.3	1390.6*# ± 110.1
**Cardiac tissue**	544.3 ± 63.0	739.5* ± 82.3	1060 * ± 90.5	1593.7* § ± 117.8	1206*§# ± 148.4	793.7*§ ± 125.8	1393.7* §#• ± 214.5

Results are presented as mean ± SD.

*Values differ significantly from control (p < 0.05). #Values differ significantly from Diabetic + NCD (p < 0.05).

^§^Values differ significantly from diabetic animals (p < 0.05). • Values differ significantly from Diabetic + ZnPP (p < 0.05).

A significant increase (p < 0.001) in the HO activity in the pancreas and the heart was detected in diabetic rats receiving pure curcumin when compared with diabetic rats. Pure curcumin supplementation showed a significant decrease (p < 0.001) in the pancreas and heart compared with the diabetic group receiving the oral NCD derivative (Table 5).

### HO-1 gene expression in heart and pancreas

There was a significant increase (p < 0.001) in mean HO-1 expression level in the pancreas (0.95 ± 0.04 HO-1/β-actin) and heart (0.80 ± 0.06 HO-1/β-actin) of diabetic rats compared with the corresponding control groups (0.32 ± 0.02 HO-1/β-actin and 0.24 ± 0.02 HO-1/β-actin), respectively.

NCD supplementation to the control rats significantly increased (p < 0.001) HO-1 expression in the pancreas (0.58 ± 0.04 HO-1/β-actin) and heart (0.55 ± 0.06 HO-1/β-actin) compared with the corresponding control groups (0.32 ± 0.02 HO-1/β-actin and 0.24 ± 0.2 HO-1/β-actin), respectively.

In addition, NCD supplementation to the diabetic rats significantly increased (p < 0.001) HO-1 expression in the pancreas (1.86 ± 0.06 HO-1/β-actin), and heart (1.37 ± 0.05 HO-1/β-actin) compared with the corresponding control groups (0.32 ± 0.02 HO-1/β-actin and 0.24 ± 0.02 HO-1/β-actin) and corresponding diabetic groups (0.95 ± 0.03 HO-1/β-actin and 0.80 ± 0.06 HO-1/β-actin), respectively.

**Figure 1:**
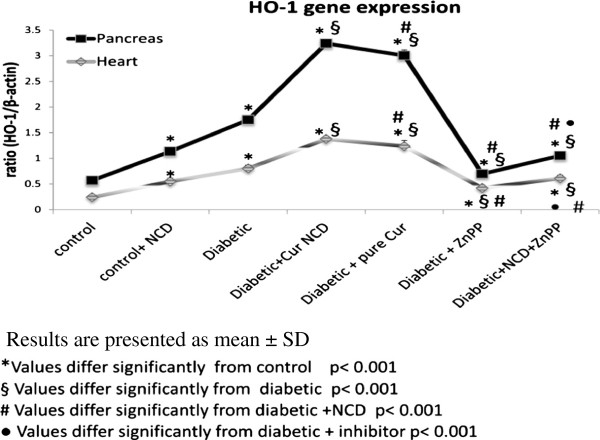
**Comparison between the heme oxygenase-1 gene expression of studied groups. **Results are presented as mean ± SD. *Values differ significantly from control p < 0.001. §Values differ significantly from diabetic p < 0.001. #Values differ significantly from diabetic + NCD p < 0.001. ^• ^Values differ significantly from diabetic + inhibitor p < 0.001.

A significant increase (p < 0.001) in the HO-1 gene expression in the pancreas (1.76 ± 0.03 HO-1/β-actin) and the heart (1.23 ± 0.1 HO-1/β-actin) was detected in diabetic rats receiving pure curcumin when compared with diabetic rats (0.95 ± 0.03 HO-1/β-actin) and (0.95 ± 0.03 HO-1/β-actin), respectively. Whereas, pure curcumin supplementation showed a significant decrease (p < 0.001) compared with diabetic group receiving the oral NCD derivative in the pancreas (1.86 ± 0.06 HO-1/β-actin) and heart (1.37 ± 0.05 HO-1/β-actin) (Figure 1).

### p300 gene expression in the heart

There was a significant increase (p < 0.001) in the mean p300 gene expression level in the heart (0.85 ± 0.08 p300/β-actin) of diabetic rats compared with the control group (0.24 ± 0.02 p300/β-actin). On the other hand mean p300 gene expression level in the control group (0.24 ± 0.02 p300/β-actin) showed no significant difference (p > 0.05) compared with the control group receiving the oral NCD (0.22 ± 0.04 p300/β-actin).

In addition, NCD supplementation to the diabetic rats significantly decreased (p < 0.001) p300 gene expression in the heart (0.48 ± 0.04 p300/β-actin) when compared with the diabetic group (0.85 ± 0.08 p300/β-actin).

**Figure 2:**
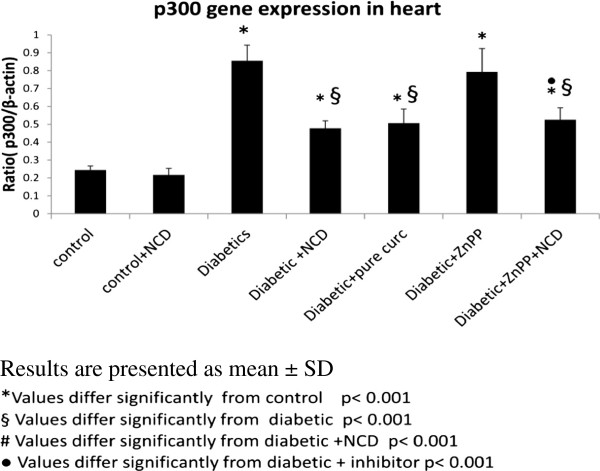
**Comparison between the p300 gene expression of studied groups. **Results are presented as mean ± SD. *Values differ significantly from control p < 0.001. §Values differ significantly from diabetic p < 0.001. #Values differ significantly from diabetic + NCD p < 0.001. ^• ^Values differ significantly from diabetic + inhibitor p < 0.001.

A significant decrease (p < 0.001) in the mean p300 gene expression level in the heart of the diabetic group receiving pure curcumin (0.51 ± 0.07 p300/β-actin) compared with the diabetic group (0.85 ± 0.08 p300/β-actin) was detected. Whereas, it showed no significant difference (p > 0.05) compared with the diabetic group receiving the oral NCD (0.48 ± 0.04 p300/β-actin) (Figure 2).

### Gene expression of molecular markers of cardiac hypertrophy

There was a significant increase (p < 0.001) in the mean ANP (0.68 ± 0.13 ANP/β-actin), MEF2A (2.52 ± 0.14 MEF2A/β-actin) and MEF2C (1.81 ± 0.12 MEF2C/β-actin) gene expression levels in the heart of diabetic rats compared to the corresponding control groups (0.026 ±0.01ANP/β-actin), (0.18 ± 0.02 MEF2A/β-actin) and (0.15 ± 0.01 MEF2C/β-actin), respectively.

On the other hand the mean ANP (0.024 ± 0.01 ANP/β-actin), MEF2A (0.17 ± 0.02 MEF2A/β-actin) and MEF2C (0.16 ± 0.01MEF2C/β-actin) gene expression levels in the control groups receiving the NCD showed no significant difference (p > 0.05) compared with the corresponding control groups (0.026 ±0.01ANP/β-actin), (0.18 ± 0.0 MEF2A/β-actin) and (0.15 ± 0.01 MEF2C/β-actin), respectively.

In addition, NCD supplementation to the diabetic rats significantly decreased (p < 0.001) the gene expression of ANP (0.224 ± 0.09 ANP/β-actin), MEF2A (0.998 ± 0.09 MEF2A/β-actin), MEF2C (0.85 ± 0.09 MEF2C/β-actin) when compared with the corresponding diabetic groups (0.68 ± 0.13 ANP/β-actin), (2.52 ± 0.14 MEF2A/β-actin and 1.81 ± 0.12 MEF2C/β-actin), respectively.

**Figure 3:**
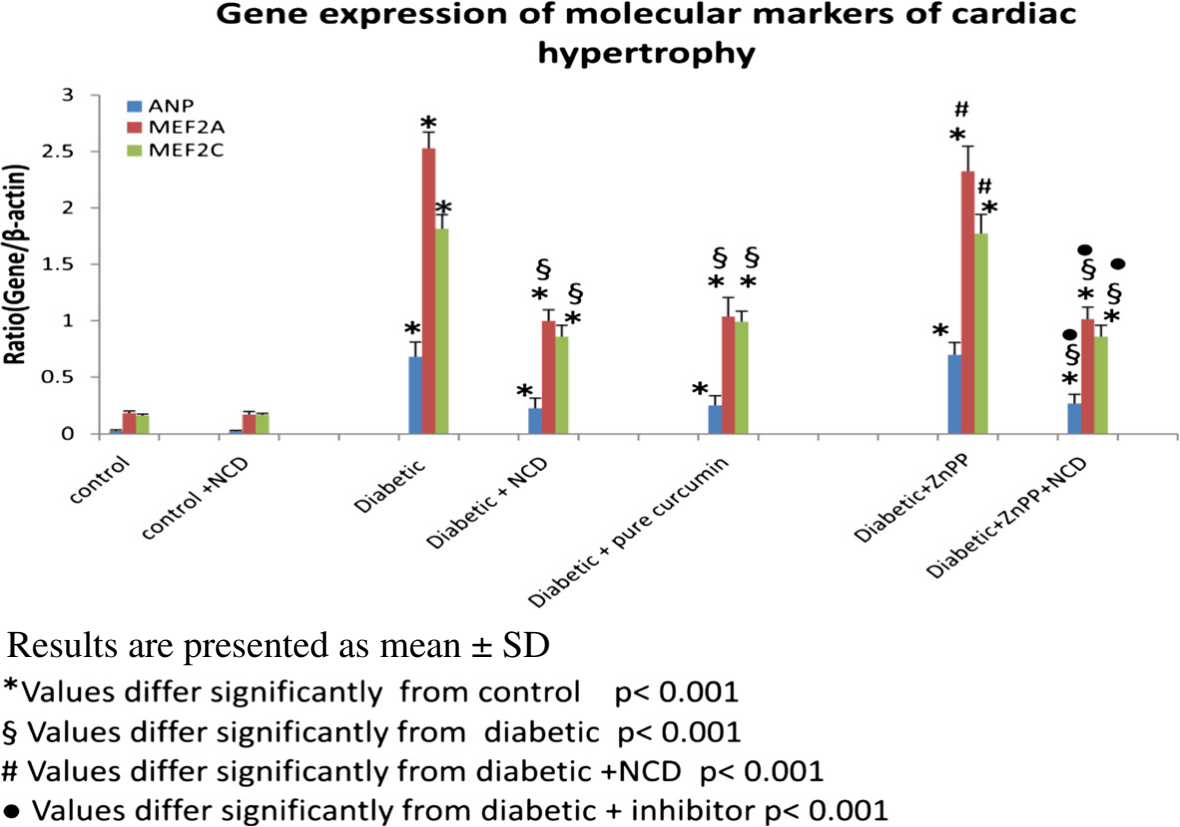
**Comparison between the gene expression of molecular markers of studied groups. **Results are presented as mean ± SD. *Values differ significantly from control p < 0.001. §Values differ significantly from diabetic p < 0.001. #Values differ significantly from diabetic + NCD p < 0.001. ^• ^Values differ significantly from diabetic + inhibitor p < 0.001.

A significant decrease (p < 0.001) in the mean ANP (0.249 ± 0.08 ANP/β-actin), MEF2A (1.03 ± 0.17 MEF2A/β-actin) and MEF2C (0.99 ± 0.1 MEF2C/β-actin) the heart of the diabetic group receiving pure curcumin compared with the diabetic groups (0.68 ± 0.13 ANP/β-actin), (2.52 ± 0.14 MEF2A/β-actin) and (1.81 ± 0.12 MEF2C/β-actin), respectively was detected. Whereas, it showed no significant difference (p > 0.05) compared with the diabetic group receiving the oral NCD, (0.224 ± 0.09 ANP/β-actin), (0.99 ± 0.09 MEF2A/β-actin) and (0.85 ± 0.09 MEF2C/β-actin), respectively (Figure 3).

### The effect of HO inhibitor (ZnPP IX) on the studied parameters

In the present study there was no significant difference in the mean heart rate, SBP, LVDP and LV dp/dt in the diabetic group receiving HO inhibitor ZnPP compared with the diabetic group indicating the absence of any effect of HO-1 on these parameters (Table 3).

In the diabetic group receiving the HO inhibitor ZnPP, the mean plasma glucose level showed a significant increase (p < 0.05), while the mean fasting plasma insulin level showed a significant decrease (p < 0.05) compared with the diabetic group (Table 4).

The mean plasma glucose level in the diabetic group receiving the oral NCD combined with HO inhibitor ZnPP showed a significant increase (p < 0.01), while the mean fasting plasma insulin level showed a significant decrease (p < 0.05) when compared with the diabetic group receiving the oral water soluble curcumin derivative (Table 4).

In addition, administration of ZnPP to diabetic rats or diabetic rats receiving NCD showed no significant difference (p > 005) in mean GHb level compared with the diabetic group (Table 4).

Administration of ZnPP to diabetic rats significantly decreased (p < 0.001) HO-activity (Table 5) and expression in the pancreas and heart (Figure 1) compared with the corresponding diabetic groups.

Administration of ZnPP to diabetic rats or diabetic rats receiving NCD showed no significant difference (p > 0.05) in the mean p300 (Figure 2), and ANP, MEF2A and MEF2C (Figure 3) gene expression levels and in the heart of the diabetic group and the diabetic group receiving oral NCD, respectively.

## Discussion

Several studies reported that curcumin treatment induced hypoglycemia in rats with STZ-induced DM [23,24]. In the present study, administration of oral NCD or pure curcumin to diabetic rats significantly decreased blood glucose levels and increased the plasma insulin compared with the diabetic group. However, their levels did not reach those of normal controls. Diabetic rats receiving pure curcumin showed no significant difference in the plasma glucose level compared with the diabetic group receiving the NCD.

The results of the current study are in accordance with the work of others who reported that administration of oral curcumin to diabetic mice or rats fed with curcumin resulted in a significant decrease in the blood glucose level when compared with the diabetic group [25,26].

In the present work, oral NCD did not change the plasma glucose levels in the control group while it significantly increased the plasma insulin in the control group. This agrees with another study [27] where the ingestion of 6 g *C. longa* increased postprandial serum insulin levels, but did not affect plasma glucose levels in healthy subjects. Therefore, *C. longa* may have an effect on insulin secretion. They pointed to the fact that in healthy subjects, glucose levels are strictly regulated and it is difficult to measure differences in plasma glucose levels.

Several mechanisms may explain how curcumin mediates its hypoglycemic effects. Best et al. [28] reported that curcumin activated the volume-regulated anion channels in β-cells which was accompanied by the depolarization of the cell membrane potential, the generation of electrical activity, and enhanced insulin secretion. Another report indicated that curcumin treatment enhances islet recovery by inducing heat-shock protein Hsp70, a response protein, during cryopreservation [29] so the activation of β-cells function by curcumin might contribute to the hypoglycemic actions of this compound.

The current study agrees with the results of our previous work [6] on the effect of curcumin on insulin release in rat isolated pancreatic islets. We reported that insulin secretion was significantly increased in islets incubated with curcumin. Insulin secretion was significantly decreased by incubation of islets with stannus mesoporphyrin (HO activity inhibitor), indicating the role of HO-1 in insulin secretion in pancreatic islets. Additionally, curcumin was found to induce HO-1 expression, which has been reported to have cytoprotective effects in mouse pancreatic beta-cells [5].

To determine whether curcumin’s action on blood glucose and insulin was mediated via inducible HO-1, the HO-1 inhibitor (ZnPP) was administered to diabetic rats and diabetic rats receiving oral NCD. This resulted in a significant increase in the plasma glucose level and a significant decrease in insulin levels when compared with the diabetic group receiving oral NCD only. Also, the diabetic rats receiving ZnPP only demonstrated significantly higher glucose levels and lower insulin levels compared with the diabetic rats. This suggests that the hypoglycemic action of curcumin may be, in part, mediated through HO-1.

In the present work, there was a significant increase in the mean GHb level in the diabetic group compared with the controls. Diabetic rats supplemented with the NCD or pure curcumin showed a modest but significantly lower blood GHb level compared with the diabetic group. But, their levels were still higher than the control group and no significant difference was detected between diabetic rats receiving the oral NCD or pure curcumin. Several studies are consistent with these results [30,31].

In the present study, there was a significant increase in the mean HO-1 expression level and activity in the pancreatic and cardiac tissues of the diabetic group compared with controls. The effect of diabetic conditions on HO-1 expression and activity has been investigated in various models in vitro and in vivo and the results of those experiments are inconsistent [32,33]. However, even if regulation of HO-1 expression in DM is still uncertain, doubtlessly hyperglycemia leads to endothelial dysfunction, impaired cell replication and increased apoptosis [34] and these effects are reversed by overexpression of anti-oxidative enzymes, such as HO-1 [35]. DM caused up-regulated expression of HO-1 in pancreatic β-cells of ob/ob mice [36] and db/db mice [37], in liver [38] and in glomerular cells of diabetic rats [32].

The present study showed that oral supplementation of the NCD to control and diabetic rats, significantly increased HO-1 expression and activity in the cardiac tissue of the diabetic group compared with the controls. In accordance with our results, HO mRNA, protein (both isozymes) and activity was up-regulated in the heart of diabetic rats [8].

Diabetic cardiomyopathy is characterized functionally by ventricular dilation, myocyte hypertrophy, prominent interstitial fibrosis and decreased or preserved systolic function [39] in the presence of a diastolic dysfunction [40]. In the present study, there was a significant decrease in the heart rate, LVDP, LV dp/dt and a significant increase in systolic blood pressure in diabetic rats compared with the control group.

The present results, agrees with the work of Radovits et al. [41] and Riad et al. [42] who reported a significant decrease in HR, LVDP and LV dp/dt in diabetic rats indicating a decrease in cardiac contractility.

In diabetic rats receiving the NCD or pure curcumin, left ventricular function was improved as indicated by increased heart rate, LVDP, LV dp/dt and decreased systolic blood pressure when compared with diabetic rats. This agrees with the results of Connelly et al. [43] who reported that curcumin-treated diabetic rats demonstrated reduced cardiac hypertrophy, improved chamber compliance and enhanced systolic function when compared with untreated diabetic counterparts.

In the present work, diabetic rats receiving pure curcumin showed no significant difference compared with the diabetic group receiving the NCD. Also, feeding NCD did not affect these parameters in the control group.

HO-1 is a very effective defensive system against oxidative stress-induced cardiac cell damage [44] as well as cardiac ischemia-reperfusion injury and organ rejection [45,46]. In addition, both CO and bilirubin, the 2 major products of heme degradation by HO, have been demonstrated to exert a direct and significant protective effect both in cardiac tissue [46] and during cold preservation of organs [47].

Atrial and brain natriuretic peptides (ANP and BNP, respectively) are polypeptide hormones comprising the cardiac-derived natriuretic peptide system [48,49]. ANP is usually synthesized in the atria, while BNP is primarily synthesized in the ventricles.

After birth, ventricular expression of both ANP and BNP is upregulated in several pathological conditions of the heart, and their plasma concentrations are markedly elevated in patients with cardiac hypertrophy or congestive heart failure (CHF) [50].

MEF2 is an important transcription factor in myocyte hypertrophy [12]. MEF2 is associated with class II HDACs. Translocation of HDAC to the cytoplasm frees up MEF2, which allows for its association with HATs, like p300, leading to the transcription of effector genes [12,13]. MEF2 controls the expression of many fetal cardiac genes. The normal adult heart has no MEF2-dependent gene expression [51]. However, MEF2 gene expression is activated in cardiac hypertrophy. Moreover, blockade of MEF2-dependent gene expression completely inhibits cardiac hypertrophy caused by a variety of prohypertrophic stimuli [52].

In the present study, ANP, MEF2A and MEF2C (molecular markers of cardiac hypertrophy) gene expressions were significantly higher in the diabetic group compared with the controls. However, in diabetic rats receiving NCD or pure curcumin the gene expression of ANP, MEF2A and MEF2C was significantly lower than the diabetic group. In addition, the p300 gene expression showed similar results.

Our results agree with several studies that reported that the expression and secretion of ANP and BNP is up-regulated in diseased hearts such as those showing cardiac hypertrophy or cardiomyopathy, and mechanical stress stimulates the synthesis and secretion of ANP and BNP in both atrial and ventricular cells [53,54].

The results of the current study are in accordance with the work of Feng et al. [55] who reported that treatment with p300 blocker, curcumin prevented diabetes-induced upregulation of these transcripts suggesting the existence in curcumin of a novel glucose-induced epigenetic mechanism regulating gene expression and cardiomyocyte hypertrophy in DM.

The increase in p300 gene expression in hearts of diabetic rats may be due to hyperglycemia. Several signaling mechanisms activated by hyperglycemia may stimulate the activation of p300 [55]. It was previously demonstrated that, in endothelial cells, hyperglycemia-induced protein kinase C and mitogen-activated protein kinase, as well as protein kinase B activation, may enhance the activity of p300 [56]. All such pathways have been demonstrated in the heart of diabetic animals and may potentially contribute to p300 activation [57,58]. These studies support the notion that p300 may represent a common final pathway upon which several signaling mechanisms may converge.

Diabetic rats receiving pure curcumin showed no significant difference in ANP, MEF2A, MEF2C and p300 gene expression compared with diabetic group receiving the NCD. Also, feeding NCD did not affect these parameters in the control group.

In the present study, to determine whether inducible HO-1 in the heart contributes to the inhibitory effect of curcumin, ZnPP (HO -1 inhibitor) was administered to the diabetic rats. No significant difference was detected in physiological parameters compared with the diabetic group. Similarly, no significant difference in p300 gene expression was detected in cardiac tissues compared with the diabetic group. Also, there was no significant difference was detected in p300 gene expression in the diabetic group receiving the NCD when compared with the diabetic group receiving NCD combined with HO inhibitor ZnPP. Thus, curcumin decreased the p300 gene expression in cardiac tissue by a direct effect rather than induction of HO-1. In addition, ANP, MEF2A and MEF2C gene expressions showed similar results.

In the present study, there was no significant difference between administration of NCD and pure curcumin. However, the effect of NCD by its small dose (20 mg/Kg/day orally for 45 days) taking into consideration that the novel curcumin derivative has only a 3.0% curcumin content, gave the same results as pure curcumin (20 mg/Kg/day orally for 45 days). Thus, NCD was absorbed at a higher rate than pure curcumin and NCD still retains the essential potencies of natural curcumin.

In conclusion, NCD and curcumin decreased plasma glucose, GHb and increased insulin levels significantly in diabetic rats and its action may be partially mediated by induction of HO-1. HO-1 gene expression and HO activity were significantly increased in diabetic heart and pancreas. Diabetes upregulated expression of cardiomyopathy markers and p300. However, NCD and curcumin prevented DM-induced upregulation of these parameters and improved left ventricular function. The effect of the NCD was better than the same dose of curcumin.

## Competing interests

The authors declare no competing interest with respect to the authorship and/or publication of this article.

## Authors’ contributions

MT contributes in study design, manuscript drafting and critical discussion. IN contributes in study design, and critical discussion .DP contributes in study design, manuscript drafting and critical discussion. AR contributes in preparation of the novel curcumin derivative.MA contributes in analysis and manuscript drafting. HHA contributes in study design, practical work, manuscript drafting and critical discussion. HHF contributes in analysis and manuscript drafting.DS contributes in practical work. HM contributes in practical work, manuscript drafting and critical discussion. RE contributes in practical work, manuscript drafting and critical discussion. All authors read and approved the final manuscript.

## Authors’ information

Co-authors: Ibrahim Naguib El Ibrashy, Dimitri P Mikhailidis, Ameen Mahmoud Rezq, Mohamed Abdel Aziz Wassef, Hanan Hassan Fouad, Hanan Hosni Ahmed, Dina Sabry, Heba Mohamed Shawky, Rania Elsayed Hussein.
